# Clinicopathological Characteristics and Postoperative Outcomes of Patients Undergoing Modified Radical Mastectomy: A Retrospective Study

**DOI:** 10.7759/cureus.101373

**Published:** 2026-01-12

**Authors:** Fatima Sikandar, Sumbal Zahid, Abdul Basit Ali, Amara Younas, Kaushalendra Mani Tripathi, Paras Fatima, Filza Fatima

**Affiliations:** 1 Internal Medicine, Mid and South Essex Foundation, Essex, GBR; 2 General Surgery, Sir Ganga Ram Hospital, Lahore, PAK; 3 General Surgery, Shalamar Hospital, Lahore, PAK; 4 Internal Medicine, White River Medical Center-White River Health, Batesville, USA; 5 General Surgery, Pakistan Navy Ship (PNS) Shifa Hospital, Karachi, PAK

**Keywords:** breast cancer, breast-conserving surgery, invasive ductal carcinoma, modified radical mastectomy, sentinel lymph node

## Abstract

Background

Breast cancer remains the most common malignancy among women worldwide and a leading cause of cancer-related mortality, particularly in developing countries where delayed presentation and limited screening facilities persist.

Objective

The objective of this study is to evaluate the clinicopathological profile and short-term (30-day) postoperative outcomes of patients with breast cancer undergoing modified radical mastectomy (MRM) in a resource-limited tertiary care setting while exploring factors that may influence surgical complications.

Methods

This retrospective observational study included 210 female patients who underwent MRM between January 2022 and December 2024. Demographic, clinical, histopathological, perioperative, and 30-day postoperative outcome data were extracted from hospital records and analyzed using SPSS version 26.0 (IBM Corp., Armonk, NY).

Results

The mean age was 51.4 ± 10.2 years, with 63.8% of patients being postmenopausal. Invasive ductal carcinoma was the predominant histological subtype (89.5%). Most patients presented with locally advanced disease (stage IIIC, 60%), and axillary lymph node involvement was observed in 65.7%. Estrogen receptor (ER) positivity was noted in 62.9%, progesterone receptor (PR) positivity in 57.1%, human epidermal growth factor receptor 2 (HER2/neu) overexpression in 25.7%, and triple-negative breast cancer in 17.1% of cases. The mean operative time was 115 ± 25 minutes, and the mean blood loss was 210 ± 60 mL. Postoperative complications occurred in 27.6% of patients, most commonly seroma formation (16.2%). No 30-day postoperative mortality was observed.

Conclusion

Modified radical mastectomy remains a safe and effective surgical option for breast cancer management in resource-limited settings, providing acceptable morbidity and reliable short-term outcomes, particularly among patients presenting with advanced disease.

## Introduction

Breast cancer is the most commonly diagnosed malignancy among women worldwide and represents a major global public health concern. According to the World Health Organization, approximately 2.3 million new cases of breast cancer are diagnosed annually, accounting for nearly 25% of all cancers in women. While breast cancer is not the leading cause of cancer-related mortality globally, it remains the leading cause of cancer death among women in many low- and middle-income countries, largely due to delayed diagnosis and limited access to comprehensive treatment facilities [1].

Breast cancer is a heterogeneous disease with diverse histological and molecular subtypes that influence clinical behavior and therapeutic strategies. The most common histological subtype is invasive ductal carcinoma, followed by invasive lobular carcinoma and other less frequent variants. Tumor biology is further characterized by hormone receptor status, including estrogen receptor (ER), progesterone receptor (PR), and human epidermal growth factor receptor 2 (HER2/neu), which play a critical role in prognosis and treatment planning. Triple-negative breast cancer, lacking ER, PR, and HER2 expression, is associated with more aggressive disease and poorer outcomes, often necessitating more extensive surgical and systemic treatment approaches [2].

Early diagnosis through screening and timely intervention allows for breast-conserving surgery (BCS) combined with sentinel lymph node biopsy and adjuvant radiotherapy, which constitutes the standard of care in early-stage breast cancer. However, in low-resource settings such as Pakistan, limited screening programs, a lack of awareness, sociocultural barriers, and financial constraints frequently result in delayed presentation. Consequently, a large proportion of patients present with locally advanced disease, for which modified radical mastectomy (MRM) remains the most commonly employed surgical treatment [3].

Modified radical mastectomy, first described by Patey and Dyson as a refinement of Halsted’s radical mastectomy, involves the complete removal of breast tissue with axillary lymph node dissection while preserving the pectoralis major muscle. This technique aims to achieve adequate oncologic clearance while reducing surgical morbidity. Despite global shifts toward breast conservation, MRM continues to play a vital role in the management of locally advanced, multifocal, and biologically aggressive breast cancers, particularly in settings where access to radiotherapy, reconstructive surgery, and multidisciplinary oncologic care is limited [4].

Several patient-related and tumor-related factors influence treatment selection and postoperative outcomes in breast cancer. Sociodemographic variables such as age at diagnosis, menopausal status, parity, family history, and comorbid conditions, including obesity, diabetes mellitus, and hypertension, have been shown to affect disease presentation, surgical morbidity, and recovery. Tumor-specific factors, such as tumor size, histological grade, lymphovascular invasion, axillary lymph node involvement, and hormone receptor status, play a decisive role in determining the extent of surgery and the need for adjuvant therapy [5-7].

Long-term outcomes in breast cancer are strongly influenced by the adequacy of surgical margins, extent of lymphatic clearance, and integration of multimodal treatment strategies, including chemotherapy, hormonal therapy, radiotherapy, and targeted therapy. Although advances in systemic therapy have significantly improved survival and reduced recurrence rates, the absence of structured multidisciplinary follow-up and limited access to adjuvant treatments in developing regions continue to compromise optimal outcomes [8].

Socioeconomic and cultural factors further contribute to delayed healthcare-seeking behavior. Fear, social stigma, the lack of education, and financial hardship often result in postponed presentation and advanced-stage disease at diagnosis, necessitating radical surgical interventions. In several regions of South Asia, insufficient community-based screening programs and inadequate public health infrastructure highlight the urgent need for effective awareness campaigns and early detection strategies to reduce the burden of advanced breast cancer [9-11].

This study aimed to evaluate the clinicopathological characteristics and postoperative outcomes of patients with breast cancer undergoing modified radical mastectomy at a tertiary care hospital, providing insight into disease patterns and surgical outcomes in a resource-limited setting.

## Materials and methods

Methodology

This study was designed as a retrospective observational analysis conducted at the Department of General Surgery, Shalamar Hospital, Lahore, Pakistan, a tertiary care teaching hospital managing a high volume of breast cancer cases annually. The study period extended from January 2022 to December 2024. The hospital provides comprehensive oncologic surgical services, including modified radical mastectomy, and maintains detailed electronic and physical medical records. Ethical approval for this study was obtained from the Institutional Review Board/Ethics Committee of Shalamar Hospital, Lahore. Given the retrospective nature of the study and the use of anonymized patient data, the requirement for informed consent was waived by the Ethics Committee. All procedures were conducted in accordance with the ethical principles outlined in the Declaration of Helsinki.

Study population

A total of 210 female patients diagnosed with invasive breast cancer who underwent modified radical mastectomy during the study period were included. Patient eligibility was determined based on demographic, clinical, operative, and histopathological records documented in the hospital archives.

Inclusion and exclusion criteria

The study included female patients aged 18 years and above who were diagnosed with invasive breast cancer, including ductal, lobular, or other histological subtypes, and who underwent modified radical mastectomy. Only patients with complete and accessible medical records were eligible for inclusion to ensure the accuracy and reliability of extracted clinical data.

Patients were excluded if they were male or younger than 18 years, presented with metastatic disease at the time of diagnosis, or underwent surgical procedures other than modified radical mastectomy, such as breast-conserving surgery or alternative operative approaches. Additionally, cases with incomplete documentation or unavailable follow-up records were excluded to avoid data gaps that could compromise the validity of outcome assessment.

Data collection

Data were collected retrospectively using a structured data extraction proforma developed specifically for this study. Patient records were reviewed from the hospital’s electronic medical record system, operative theatre logs, histopathology reports, and follow-up files. The variables collected included demographic information (age, marital status, parity, menopausal status, family history, and comorbidities), clinical features (presenting symptoms, side of involvement, tumor size, and clinical lymph node status), perioperative parameters (operative duration, intraoperative blood loss, number of lymph nodes removed, drain duration, and length of hospital stay), histopathological data (tumor subtype, grade, lymphovascular invasion, and hormone receptor status including ER, PR, and HER2/neu), and 30-day postoperative outcomes, including complications such as seroma, hematoma, flap necrosis, surgical site infection (SSI), lymphedema, and mortality.

Postoperative complications

Postoperative complications included the following: seroma: clinically palpable fluid collection at the surgical site confirmed by aspiration if necessary; hematoma: localized collection of blood at the operative site requiring intervention or observation; flap necrosis: any area of skin flap showing full-thickness necrosis or requiring debridement; surgical site infection (SSI): redness, swelling, purulent discharge, or systemic signs within 30 days post-surgery, classified per CDC criteria; lymphedema: swelling of the ipsilateral arm appearing after surgery, confirmed by clinical examination; and mortality: death occurring within 30 days postoperatively.

Tools and guidelines used

Tumor staging was performed according to the AJCC Cancer Staging Manual, Eighth Edition, which is freely available for clinical use [12]. Hormone receptor and HER2/neu status were interpreted following ASCO/CAP guidelines, which are publicly available and require no licensing fees [13]. Tumor grading was based on the Nottingham Histologic Grading System, also free for clinical and research use [14]. These tools and guidelines were cited appropriately in the manuscript and referenced in figures and tables where applicable. Interobserver variability for histopathological assessment was minimized by having all slides reviewed by two independent pathologists, and discrepancies were resolved by consensus.

Statistical analysis

All data were analyzed using SPSS version 26.0 (IBM Corp., Armonk, NY). Continuous variables were expressed as mean ± standard deviation (SD), while categorical variables were presented as frequencies and percentages. Associations between clinicopathological variables and postoperative complications were evaluated using the chi-square test when expected cell counts were sufficient or Fisher’s exact test when expected frequencies were <5. Continuous variables between two groups were compared using the independent samples t-test. A p-value of <0.05 was considered statistically significant. Missing data were handled by excluding cases from specific analyses if records were incomplete, and this is acknowledged as a limitation of the study.

## Results

The study included 210 patients with a mean age of 51.4 ± 10.2 years. Most patients belonged to the 41-60-year age group (132, 62.8%), followed by 21-40 years (48, 22.9%) and >60 years (30, 14.3%). A majority of patients were postmenopausal (134, 63.8%), indicating higher disease prevalence in older women. A positive family history of breast cancer was reported in 28 (13.3%), while 182 (86.7%) had sporadic disease. Hypertension was present in 75 (35.7%), diabetes mellitus in 61 (29.0%), and both conditions in 34 (16.2%) patients (Table 1). Body mass index (BMI) and smoking history were inconsistently documented in retrospective records and therefore were not included in the formal analysis. Their potential impact on surgical outcomes is acknowledged as a limitation.

**Table 1:** Demographic Characteristics of the Study Participants (n = 210) Data are presented as frequency (n) and percentage (%) or mean ± standard deviation (SD). Data presented descriptively; no inferential statistics were applied

Variables	Values (n, %/Mean ± SD)
Age 21-40 Years	48 (22.9%)
Age 41-60 Years	132 (62.8%)
Age > 60 Years	30 (14.3%)
Mean Age (Years)	51.4 ± 10.2
Premenopausal	76 (36.2%)
Postmenopausal	134 (63.8%)
Family History Present	28 (13.3%)
Family History Absent	182 (86.7%)
Hypertension (HTN)	75 (35.7%)
Diabetes Mellitus (DM)	61 (29.0%)
Both HTN and DM	34 (16.2%)
No Comorbidity	40 (19.1%)

A painless breast lump was the most frequent presenting symptom (196, 93.3%). The right breast was involved in 114 (54.3%) cases. Clinical axillary lymphadenopathy was detected in 141 (67.1%), reflecting advanced disease at presentation. The mean tumor size was 4.2 ± 1.3 cm. Based on AJCC Eighth Edition staging, 126 (60%) presented with stage III disease, 42 (20%) with stage II, and 42 (20%) with stage IIIC (locally advanced disease). No patients with distant metastatic (true stage IV) disease were included. Histopathologically, invasive ductal carcinoma (188, 89.5%) was predominant (Table 2).

**Table 2:** Clinical and Tumor Characteristics of the Patients (n = 210) Data are presented as frequency (n) and percentage (%) or mean ± SD. Descriptive statistics only; no inappropriate p-values reported *Tumor staging was performed according to the AJCC Cancer Staging Manual, Eighth Edition [12]. Tumor grading was based on the Nottingham Histologic Grading System [14] SD: standard deviation

Variables	Values (n, %/Mean ± SD)
Breast Lump	196 (93.3%)
Nipple Retraction	38 (18.1%)
Skin Dimpling	26 (12.4%)
Ulceration	10 (4.8%)
Right Breast	114 (54.3%)
Left Breast	96 (45.7%)
Clinical Axillary Lymphadenopathy	141 (67.1%)
Tumor Size (cm)	4.2 ± 1.3
Stage II*	42 (20.0%)
Stage III*	126 (60.0%)
Stage IIIC (Locally Advanced)*	42 (20.0%)
Invasive Ductal Carcinoma*	188 (89.5%)
Invasive Lobular Carcinoma*	15 (7.1%)
Other Variants	7 (3.3%)

Axillary lymph node involvement was present in 138 (65.7%) patients, with 84 (40%) having ≥4 positive nodes. The mean number of nodes dissected was 15.4 ± 3.7. ER positivity was observed in 132 (62.9%), PR in 120 (57.1%), and HER2/neu in 54 (25.7%), while triple-negative breast cancer accounted for 36 (17.1%) cases (Table 3). Triple-negative tumors were more frequently associated with stage III/IIIC disease and showed a higher incidence of postoperative complications compared to hormone-receptor-positive tumors (p = 0.043).

**Table 3:** Hormone Receptor and Nodal Status (n = 210) Data are presented as frequency (n) and percentage (%) or mean ± SD *Hormone receptor and HER2/neu status were interpreted following ASCO/CAP guidelines [13] SD, standard deviation; HER2/neu, human epidermal growth factor receptor 2; ER, estrogen receptor; PR, progesterone receptor

Variables	Values (n, %/Mean ± SD)
Axillary Node Involvement	138 (65.7%)
1-3 Nodes Positive	54 (25.7%)
≥4 Nodes Positive	84 (40.0%)
Mean Nodes Dissected	15.4 ± 3.7
ER-Positive*	132 (62.9%)
PR-Positive*	120 (57.1%)
HER2/Neu-Positive*	54 (25.7%)
Triple-Negative*	36 (17.1%)

All surgeries were performed or directly supervised by consultant general surgeons with ≥10 years of post-fellowship experience in breast surgery, ensuring procedural standardization. The mean operative time was 115 ± 25 minutes, and the mean blood loss was 210 ± 60 mL. Postoperative complications occurred in 58 (27.6%) patients, with seroma being the most frequent (34, 16.2%) (Table 4).

**Table 4:** Surgical and Postoperative Outcomes (n = 210) Continuous variables are presented as mean ± SD and categorical variables as frequency (n) and percentage (%) SD: standard deviation

Variables	Values (n, %/Mean ± SD)
Operative Time (Minutes)	115 ± 25
Blood Loss (mL)	210 ± 60
Hospital Stay (Days)	6.2 ± 2.1
Drain Duration (Days)	7.3 ± 2.4
Any Complications	58 (27.6%)
Seroma	34 (16.2%)
Wound Infection	14 (6.7%)
Flap Necrosis	6 (2.9%)
Hematoma	4 (1.9%)
Lymphedema	8 (3.8%)
30-Day Mortality	0

Complications were more frequent among older patients, with rates of 14 (46.7%) in those above 60 years compared to eight (16.7%) in patients aged ≤40 years (p = 0.041). Postmenopausal women had significantly higher complication rates (44, 32.8%) than premenopausal women (14, 18.4%) (p = 0.032). Tumor size greater than 3 cm was also significantly associated with postoperative complications (50 {34.2%} versus eight {12.5%}, p = 0.018). Similarly, patients with positive lymph nodes experienced more complications (34.8%) than those with negative nodes (10, 13.9%) (p = 0.007). The presence of comorbidities, especially diabetes and hypertension, correlated with higher complication rates (38 {34.5%} versus 20 {20.0%}, p = 0.022) (Table 5).

**Table 5:** Association Between Clinicopathological Variables and Postoperative Complications (n = 210) Data are presented as frequency (n) and percentage (%). Comparisons between groups were performed using the chi-square (χ²) test for categorical variables and Student’s t-test for continuous variables. P-values < 0.05 were considered statistically significant

Variables	Total (n)	Patients With Complications (n, %)	Test Statistics	P-values
Age ≤ 40 Years	48	8 (16.7%)	χ² = 6.21	0.041
Age 41-60 Years	132	36 (27.3%)	-	-
Age > 60 Years	30	14 (46.7%)	-	-
Premenopausal	76	14 (18.4%)	χ² = 4.58	0.032
Postmenopausal	134	44 (32.8%)	-	-
Tumor ≤ 3 cm	64	8 (12.5%)	χ² = 5.65	0.018
Tumor > 3 cm	146	50 (34.2%)	-	-
Lymph Node-Negative	72	10 (13.9%)	χ² = 7.26	0.007
Lymph Node-Positive	138	48 (34.8%)	-	-
Comorbidities Present	110	38 (34.5%)	χ² = 5.26	0.022
Comorbidities Absent	100	20 (20.0%)	-	-
Duration of Surgery < 120 Minutes	132	26 (19.7%)	χ² = 6.45	0.013
Duration of Surgery ≥ 120 Minutes	78	32 (41.0%)	-	-

## Discussion

This study evaluated the clinical characteristics and surgical outcomes of 210 patients with breast cancer who underwent modified radical mastectomy (MRM) at a tertiary care center. The majority of patients presented with advanced-stage disease, predominantly invasive ductal carcinoma, and a high prevalence of axillary lymph node involvement. These findings underscore the continued relevance of MRM as a primary surgical modality in settings where late presentation is common and access to breast-conserving surgery, radiotherapy, and reconstructive services remains limited. In low- and middle-income countries such as Pakistan, delayed diagnosis and underdeveloped screening programs necessitate reliance on more extensive surgical approaches to achieve adequate locoregional disease control.

The mean age of the patients in this study was 51.4 years, which is consistent with reports from Pakistan and other South Asian countries, where breast cancer tends to present at a younger age compared to Western populations, where the mean age at diagnosis often exceeds 60 years [15]. This younger age at presentation has been attributed to a multifactorial interplay of genetic susceptibility, reproductive factors, environmental exposures, and lifestyle patterns. Furthermore, the predominance of postmenopausal women (63.8%) in our cohort aligns with findings by Khan et al., who reported a higher disease burden among middle-aged and older women in Pakistan, emphasizing the need for targeted screening strategies in this demographic [16].

A notable finding of this study was the low prevalence of positive family history (13.3%), indicating that the majority of breast cancer cases were sporadic rather than familial. This observation is consistent with global epidemiological data, which suggest that approximately 85%-90% of breast cancers worldwide occur sporadically, while only a minority are associated with inherited genetic mutations such as *BRCA1* and *BRCA2*. Similar proportions have been reported in regional and international studies, reinforcing that population-level screening and awareness programs are likely to have a greater impact on disease burden than family-history-based surveillance alone. The predominance of sporadic cases highlights the importance of modifiable risk factor control, early symptom recognition, and community education to facilitate timely presentation and diagnosis.

Painless breast lump was the most common presenting symptom (93.3%), a finding consistent with existing literature and reflective of delayed health-seeking behavior, as early breast cancer is often asymptomatic. The high frequency of palpable axillary lymphadenopathy (67.1%) and the mean tumor size of 4.2 cm further indicate advanced-stage presentation at diagnosis. These patterns are likely influenced by limited screening availability, the lack of awareness, sociocultural stigma, and financial barriers to medical consultation. Invasive ductal carcinoma accounted for 89.5% of cases, mirroring tumor biology reported across Asian and Western populations, suggesting that the aggressive presentation is driven more by delayed diagnosis than by unique histological differences [17].

Hormone receptor analysis revealed ER positivity in 62.9% and PR positivity in 57.1% of patients, while 25.7% demonstrated HER2/neu overexpression [18]. These proportions are comparable to regional data and support the applicability of standard systemic treatment protocols in this population. The high rate of axillary lymph node metastasis (65.7%) reflects substantial tumor burden and reinforces the prognostic importance of axillary clearance, particularly in resource-limited settings where sentinel lymph node biopsy is not universally available. Accurate nodal staging remains critical for guiding adjuvant therapy decisions and estimating disease prognosis.

The overall postoperative complication rate of 27.6% observed in this study falls within the acceptable range reported in the literature. Seroma formation (16.2%) was the most frequent complication, consistent with reported rates of 10%-25% following MRM [19-22]. Seroma development has been associated with the extent of axillary dissection, drain duration, and patient-related factors such as body mass index. Lower rates of wound infection and flap necrosis suggest adherence to standardized surgical techniques and perioperative care protocols. Lymphedema, observed in 3.8% of patients, was primarily associated with extensive nodal dissection and prolonged drainage, underscoring the importance of postoperative physiotherapy and patient education to mitigate long-term morbidity.

The mean operative time (115 minutes) and hospital stay (6.2 days) were comparable to findings from similar institutions, reflecting procedural efficiency and surgical expertise. Importantly, the absence of 30-day mortality highlights the safety of MRM when performed by experienced surgeons in appropriately selected patients. These findings reinforce the role of MRM as a reliable and effective surgical option in advanced breast cancer, particularly in health systems constrained by limited resources [23].

This study has several limitations. Its retrospective design relies on the accuracy and completeness of existing medical records and may be subject to information bias. As a single-center study, the findings may not be fully generalizable to other settings. Additionally, body mass index (BMI) and smoking history were inconsistently documented, precluding their inclusion in formal analysis despite their known impact on surgical outcomes. The study also lacked stratification based on neoadjuvant therapy, which may influence tumor size, nodal status, and postoperative complications. Potential confounding factors, such as comorbidities and variations in surgeon experience, were not controlled through multivariate analysis, limiting the ability to isolate independent predictors of postoperative outcomes. Furthermore, the absence of long-term oncologic outcomes, such as recurrence rates and survival, limits conclusions to short-term postoperative results.

Despite these limitations, the study provides meaningful insights into the clinical profile, disease stage at presentation, and surgical outcomes of patients with breast cancer undergoing MRM in a tertiary care setting, emphasizing the urgent need for early detection strategies and comprehensive multidisciplinary breast cancer care.

## Conclusions

It is concluded that modified radical mastectomy (MRM) remains a safe, effective, and widely applicable surgical procedure for the management of breast cancer, especially in settings where late presentation and limited access to breast-conserving options are prevalent. The study findings revealed that the majority of patients presented with locally advanced disease, predominantly invasive ductal carcinoma, and a high incidence of axillary lymph node involvement, reflecting a pattern of delayed diagnosis common in developing countries. Postoperative outcomes were satisfactory, with a low rate of major complications and no perioperative mortality, indicating that MRM, when performed by skilled surgeons with proper perioperative care, provides reliable disease control and favorable short-term results.
